# *PAX6*, brain structure and function in human adults: advanced MRI in aniridia

**DOI:** 10.1002/acn3.297

**Published:** 2016-04-12

**Authors:** Mahinda Yogarajah, Mar Matarin, Christian Vollmar, Pamela J. Thompson, John S. Duncan, Mark Symms, Anthony T. Moore, Joan Liu, Maria Thom, Veronica van Heyningen, Sanjay M. Sisodiya

**Affiliations:** ^1^ Department of Clinical and Experimental EpilepsyUCL Institute of NeurologyNational Hospital for Neurology and NeurosurgeryLondonWC1N 3BGUnited Kingdom; ^2^ UCL Institute of Ophthalmology and Moorfields Eye HospitalLondonUnited Kingdom; ^3^ Division of NeuropathologyUCL Institute of NeurologyNational Hospital for Neurology and NeurosurgeryLondonUnited Kingdom; ^4^ MRC Human Genetics UnitIGMMUniversity of EdinburghCrewe RoadEdinburghEH4 2XUUnited Kingdom; ^5^ Epilepsy SocietyChalfont‐St‐PeterBucksSL9 0RJUnited Kingdom; ^6^ Present address: St George's University Hospitals NHS Foundation TrustLondonUnited Kingdom; ^7^ Present address: Department of OphthalmologyUniversity of CaliforniaSan FranciscoCalifornia; ^8^ Present address: UCL Institute of OphthalmologyLondonUnited Kingdom

## Abstract

**Objective:**

*PAX6* is a pleiotropic transcription factor essential for the development of several tissues including the eyes, central nervous system, and some endocrine glands. Recently it has also been shown to be important for the maintenance and functioning of corneal and pancreatic tissues in adults. We hypothesized that *PAX6* is important for the maintenance of brain integrity in humans, and that adult heterozygotes may have abnormalities of cortical patterning analogous to those found in mouse models.

**Methods:**

We used advanced magnetic resonance imaging techniques, including surface‐based morphometry and region‐of‐interest analysis in adult humans heterozygously mutated for *PAX6* mutations (*n* = 19 subjects and *n* = 21 controls). Using immunohistochemistry, we also studied PAX6 expression in the adult brain tissue of healthy subjects (*n* = 4) and patients with epilepsy (*n* = 42), some of whom had focal injuries due to intracranial electrode track placement (*n* = 17).

**Results:**

There were significant reductions in frontoparietal cortical area after correcting for age and intracranial volume. A greater decline in thickness of the frontoparietal cortex with age, in subjects with *PAX6* mutations compared to controls, correlated with age‐corrected, accelerated decline in working memory. These results also demonstrate genotypic effects: those subjects with the most severe genotypes have the most widespread differences compared with controls. We also demonstrated significant increases in PAX6‐expressing cells in response to acute injury in the adult human brain.

**Interpretation:**

These findings suggest a role for *PAX6* in the maintenance and consequent functioning of the adult brain, homologous to that found in other tissues. This has significant implications for the understanding and treatment of neurodegenerative diseases.

## Introduction

*PAX6* is a highly conserved transcription factor essential to the development of several tissues including the eyes, brain, and endocrine glands of vertebrates and invertebrates.^1^ In the mouse, *Pax6* is critical for survival: *Pax6* null mice die immediately after birth with absent eyes and nasal structures, and with a diencephalon that fails to innervate a severely malformed cerebral cortex.^2^ Analysis of *Small eye* mouse embryos carrying homozygous *Pax6* loss‐of‐function mutations has demonstrated the critical role of *Pax6* in the dorsoventral specification and cortical arealization of telencephalic brain regions, and its importance in cortical progenitor proliferation, and axonal migration within the brain.^3^ Only a few cases of children with mutations in both *PAX6* alleles (compound heterozygotes) have been reported, and severe eye and brain abnormalities similar to those reported in homozygote mice were found in these patients.^4^ In contrast to homozygote models, heterozygote mice have not been widely studied. In addition to the *Small eye* ocular anomalies,^5^ the olfactory bulbs of heterozygote mice are reduced in size.^6^ Heterozygous *PAX6* mutation in humans is known to cause aniridia^7^ and hyposmia or anosmia.^8^ Such parallels between human and rodent developmental anomalies suggest that aniridia presents a unique opportunity to study the multiple roles of *PAX6* in humans.

Previous magnetic resonance imaging (MRI) studies in an aniridia cohort showed unsuspected consequences of heterozygous *PAX6* mutation, including absence of the anterior commissure without callosal agenesis, absence or reduction in the olfactory bulb and the pineal gland, polymicrogyria, and an altered configuration of midline anatomy.^8^, ^9^, ^10^ In addition, functional studies revealed deficits in working memory,^11^ olfaction,^8^ and central auditory function.^12^ These observations imply developmental hypoplasia or subsequent pathological degeneration or both: the distinction is important. Recent animal studies have suggested an important role for *Pax6* in the maintenance and functioning, as well as development, of corneal,^13^, ^14^ and pancreatic tissues.^15^ A parallel role for *PAX6* in the maintenance of brain integrity in humans has major implications for our understanding of neurodegenerative disorders, and their potential treatment with stem cell‐based therapies. Furthermore, the opportunity to study structural and functional details in human cases is critical, given that the details of developmental pathways may differ in humans and mice.^16^

In this study, we hypothesized that *PAX6* is important for the maintenance of brain integrity in humans, and that adult heterozygotes may have abnormalities of cortical patterning analogous to those found in mouse models. We investigated novel cerebral MRI parameters in 19 adults with known *PAX6* mutations (Table 1), using whole brain surface‐based morphometry (SBM), and lobe‐based region‐of‐interest (ROI) analysis. Additionally, PAX6 expression was studied by immunohistochemistry in resected adult brain tissue. SBM is an unbiased, whole‐brain approach that, unlike voxel‐based morphometry (VBM), prevents the conflation of cortical area and thickness changes.^17^ Advanced quantitative postprocessing techniques provide an opportunity to extract more information from imaging data, which is important given the rarity of this condition, and the difficulty in obtaining pathological data in such a small, selected group.

**Table 1:** Demographic and genetic information on *PAX6* subjects

Subject No.	Age/gender	Mutation at DNA level	Exon (protein domain)	Protein prediction	Mutation type
1	40/F	c.115_116dupCC	5 (PD)	p.Cys40ArgfsX15	PTC by frameshift in PD predicted ‐ NMD likely
2	18/F	c.683‐6T>A	9 (HD)	p.Glu228GlyfsX5	PTC by frameshift. Exon 9 skipped (RT‐PCR). NMD likely
3	41/F	c.718C>T	9 (HD)	p.Arg240X	PTC by direct stop codon mutation – NMD likely
4	61/M	c.763C>T	9 (HD)	p.Gln255X	PTC ultimately. Mutation in last codon of exon 9 may create stop codon and/or affect splicing leading to frame shift. NMD likely
5	40/M	c.763C>T	9 (HD)	p.Gln255X	PTC ultimately. Mutation in last codon of exon 9 may create stop codon and/or affect splicing leading to frame shift. NMD likely
6	33/M	c.775dupT	10 (HD)	p.Ser259PhefsX2	PTC resulting from predicted frameshift. NMD likely
7	36/M	c.775dupT	10 (HD)	p.Ser259PhefsX2	PTC resulting from predicted frameshift. NMD likely
8	43/M	c.357 + 2dupT	6 (PD)	36 aa deletion	In‐frame deletion of 36 aa predicted in PD by RT‐PCR following splice site mutation and activation of weak exonic splice site
9	38/M	c.357 + 5G>A	6 (PD)	36 aa deletion	In‐frame deletion of 36 aa likely predicted in PD following probably splice site mutation and activation of weak exonic splice site
10	41/F	DNA mutation unknown	6 (PD)	36 aa deletion	In‐frame deletion of 36 aa predicted in PD by RT‐PCR following activation of weak exonic splice site. Actual mutation not found – may be deep intronic change
11	35/M	del exon 10 + 11	10, 11 (PST)	88 aa deletion	In‐frame deletion of 88 aa predicted as a result of deletion of exons 10 and 11 identified by MLPA
12	60/F	c.1239delT	13 (PST)	p.Asp413GlufsX*46	C‐terminal extension predicted as a result of frame‐shift beyond the NMD boundary
13	49/M	c.1267dupT	13 (PST)	p.X423LeuextX*108	C‐terminal extension predicted as a result of single‐nucleotide insertion abolishing stop codon
14	52/M	c.1267dupT	13 (PST)	p.X423LeuextX*108	C‐terminal extension predicted as a result of single‐nucleotide insertion abolishing stop codon
15	50/F	c.1267dupT	13 (PST)	p.X423LeuextX*108	C‐terminal extension predicted as a result of single‐nucleotide insertion abolishing stop codon
16	37/M	c.1267dupT	13 (PST)	p.X423LeuextX*108	C‐terminal extension predicted as a result of single‐nucleotide insertion abolishing stop codon
17	37/F	c.191G>T	6 (PD)	p.Gly64Val	Missense change predicted in PD
18	73/F	c.191G>T	6 (PD)	p.Gly64Val	Missense change predicted in PD
19	25/F	c.372C>A	6 (PD)	p.Asn124Lys	Missense change predicted in PD

PTC, premature termination codon; NMD, nonsense‐mediated decay (destruction of mRNA by cellular protection mechanism against protein fragment interference); PD, paired domain; HD, homeo domain; PST, proline serine threonine‐rich transactivation domain; aa, amino acid.

## Materials and Methods

### Subjects

Nineteen people with aniridia (aged 18–73 years, 10 men) were recruited through their attendance at a tertiary center for eye disease. Subjects have been pheno‐ and genotypically described previously^8^, ^9^, ^10^, ^11^ but underwent further clinical, imaging, and neuropsychology assessments as part of this study. Twenty‐two normal control subjects (aged 23–62 years, 11 men) were also studied for comparison. These subjects had no reported neurological or psychiatric abnormalities. The research ethics committees of the Institute of Neurology, National Hospital for Neurology and Neurosurgery, and Moorfields Eye Hospital, approved the study protocols. All subjects provided written informed consent.

### MR data acquisition

Magnetic resonance imaging studies were performed on a 3T GE Excite II scanner (General Electric, Wakashua, Milwaukee, WI). Standard imaging gradients with a maximum strength of 40 m/Tm and slew rate 150 Tm/s were used. All data were acquired using a body coil for transmission, and eight‐channel phased array coil for reception. The scanning protocol also included a coronal T1‐weighted volumetric acquisition sequence with 1.1‐mm thick slices. No parallel acquisition techniques were used.

### MR image processing

MR images were transferred in DICOM format to a dedicated Linux workstation (Centos 5). Cortical reconstruction and volumetric segmentation were performed using surface‐based morphometry (SBM) with the Freesurfer v5.1 image analysis suite (http://surfer.nmr.mgh.harvard.edu/). The technical details of SBM are described and referenced on the Freesurfer website (http://surfer.nmr.mgh.harvard.edu/). Processing includes several stages: removal of non‐brain tissue using a hybrid watershed/surface deformation procedure, automated Talairach transformation, segmentation of the subcortical white matter, deep gray and white matter volumetric structures, intensity normalization, tessellation of the gray and white matter boundaries, automated topology correction, and finally surface deformation using intensity gradients to place the gray/white and gray/cerebrospinal fluid (CSF) borders optimally at the location where the greatest shift in intensity defines the transition to the other tissue class. In this process, voxels are classified as white matter or something other than white matter based on intensity and neighbor constraints. An initial surface is then generated for each hemisphere by tiling the outside of the white matter mass for that hemisphere. This initial surface is then refined to follow the intensity gradients between the white and gray matter (this is referred to as the white surface). The white surface is then nudged to follow the intensity gradients between the gray matter and CSF (this is the pial surface). The distance between the white and the pial surfaces equates to the thickness at each location of the cortex. Surface models of the cortical surface are generated, consisting of a mesh of triangles, and the location of the mesh is controlled by adjusting the location of the vertices. A vertex is the place where the points of neighboring triangles meet and are typically about 1 mm apart. The vertex positions are adjusted such that the surface follows the contour of the maximum T1 intensity gradient between cortical white matter (WM), and cortical gray matter (GM), and between the cortical gray matter and pia. All surfaces are constructed in native anatomical space.

Following completion of the cortical models, a number of deformation procedures were performed in further data processing and analysis. These included surface inflation allowing both sulcal and gyral folds to be visualized, registration of each vertex on the inflated surface to a spherical atlas which utilized individual cortical folding patterns to match cortical geometry across subjects, parcellation of the cerebral cortex into six subject‐specific lobar regions per hemisphere based on gyral and sulcal structure, and the creation of surface‐based data including maps of cortical volume, thickness, and area. The spherical atlas naturally forms a coordinate system in which point‐to‐point correspondence between subjects can be achieved. Mean vertex‐wise thickness, area and volume differences between groups can then be displayed on the pial surface of the standard atlas. All cortical surface maps were smoothed with a 10 mm full‐width at half‐maximum surface‐based Gaussian kernel to reduce local variations in the measurements for further analysis.

### Vertex‐wise analysis

#### Cortical thickness

Representations of cortical thickness are produced using both intensity and continuity information from the entire three‐dimensional MR volume in the segmentation and deformation procedures. Thickness is calculated as the closest distance from the gray/white boundary to the gray/pial boundary at each vertex on the tessellated surface. The maps are created using spatial intensity gradients across tissue classes and are therefore not simply reliant on absolute signal intensity. The maps produced are not restricted to the voxel resolution of the original data and thus are capable of detecting submillimeter differences between groups as validated by histologic studies.^18^

#### Cortical surface area

The area assigned to each vertex on the tessellated cortical pial surface was calculated as the average area of all triangles of which the vertex is a member. The surface area of a region can be computed by adding up the area of the vertices in that region. To compare patients to controls and obtain maps of surface area alterations, we applied previously described methods.^19^ The deformation and registration of individual spheres into the common coordinate system result in a standard number of tessellations across each individual's cortical surface. However, the surface area values assigned to each vertex are redistributed to reflect the relative expansion and contraction of the cortical sheet around each vertex. This provides point‐by‐point estimates of the relative areal expansion or compression of each vertex across the entire cortical mantle in atlas space, and enables group‐wise quantification of cortical surface area.

#### Intracranial volume

An estimate of intracranial volume (ICV) was provided by FreeSurfer based on the transformation of each subject's brain into Talairach space.

### Statistical analysis

We analyzed differences in cortical pial area and thickness by computing a general linear model (GLM) of the effect of patient‐control status or group on each measure across the entire cortex at each vertex as implemented in the Query Design Estimate Contrast (QDEC) interface of FreeSurfer. Age and ICV were included as covariates in the analysis as both have been shown to correlate with thickness and surface area in both whole brain and ROI studies.^20^ We also checked for interactions between the covariates and group with regard to each dependent measure. Unless otherwise indicated, there was no interaction between group and each covariate on dependent measures.

Statistical parametric maps of significant group differences or group‐covariate interaction, were corrected for multiple comparisons using Monte Carlo simulation methods using a vertex‐wise (cluster‐forming threshold) and clusterwise threshold of *P* < 0.05. The *P*‐value for a cluster was determined through simulation in which white Gaussian noise was repeatedly synthesized on the surface, spatially smoothed, thresholded, and clustered to determine the distribution of cluster sizes under the null hypothesis. Correlation analysis of back‐normalized clusters of interest and neuropsychology scores was assessed using a Spearman correlation test because the neuropsychology data were not normally distributed.

### Cortical ROI analysis

All cortical ROI analyses were carried out using IBM SPSS Statistics for Macintosh, Version 18.0 (IBM Corp., Armonk, NY). The normality of continuous variables was assessed using the Shapiro–Wilk and Kolmogorov–Smirnov tests. The Student's *t*‐test (parametric distribution) was used to compare group differences in ICV, while the Wald–Wolfowitz runs omnibus test (nonparametric distribution) was used to compare both the location of the mean and the distribution of age within each group. The Wald–Wolfowitz runs test compares distribution locations and shapes for two groups by combining the two groups and ranking the data. Group differences in the distribution of the sexes were assessed using the chi‐squared test. A mixed design multivariate analysis of covariance (MANCOVA) was performed to investigate cortical lobar area/thickness differences between groups. In all ROI analyses, preliminary checks were conducted in order to ensure that there was no violation of the assumptions of univariate and multivariate normality, homogeneity of variances, and homogeneity of regression slopes. Where appropriate, statistical thresholds had a Bonferroni correction applied.

The dependent variables used in the cortical ROI analysis were the mean lobar area/thickness. The regions considered were the frontal, cingulum, occipital, temporal, parietal, and insula lobes, based on the Desikan–Killiany atlas as implemented in Freesurfer. The between‐subjects independent variable was patient‐control status or group, and the within‐subjects independent variable was hemisphere (right or left). The latter was used to exclude any group‐by‐side interaction. The covariates age and ICV were also included in the analysis. Hemisphere had no significant effect on cortical area or thickness, and unless otherwise indicated there was no significant interaction between hemisphere and group, nor between group and each covariate on dependent variables. Correlations between cortical thickness and age were assessed using Pearson's correlation test. All correlations are reported using two‐tailed *P*‐values.

### Neuropsychology assessment

Neuropsychological tests measured aspects of cognition associated with brain regions identified as abnormal or implicated in previous studies; they are listed below. Due to the visual deficits that are common in this patient group, only verbal measures were used.

#### Intellectual level

The vocabulary, digit span, and similarities tests from the Wechsler Adult Intelligence Scale–Revised were administered, and the scores were prorated to obtain an estimate of the participant's verbal intellectual capacity.

#### Executive functions

Subjects completed fluency measures requiring firstly the reciting of as many words beginning with the letter S (phonemic fluency) in 1 min and then as many animal names (semantic fluency) also in 1 min. The Hayling Test, a response suppression task, was administered. This requires the subject to complete two series of 15 sentences, each of which is missing the last word.^21^ In the first series, a sensible completion is required and in the second a nonsensical completion. Performance is measured in terms of mental processing speed and accuracy.

#### Memory

A prose recall task was used to assess immediate and delayed verbal recall. A verbal learning task was used to assess the ability to learn and retain a list of fifteen words over five trials. Both measures were taken from the Adult Memory and Information Processing Battery.^22^

We expected a high degree of correlation between fluency scores (semantic and phonemic fluency), and between episodic memory scores (story recall, delayed story recall, list learning, delayed list learning). Therefore, we used a principal components analysis within SPSS v18.0 to identify a factor accounting for the largest component of variance among each of these two sets of scores. By reducing these scores to a single verbal fluency score, and a single episodic memory score we were able to reduce the number of statistical correlation analyses carried out.

### Histopathologic studies

The final part of this study used immunostaining in adult human neocortex to demonstrate *PAX6* expression in both nonepilepsy controls (*n* = 4) and epilepsy surgical tissues [with gliosis only (*n* = 5), focal cortical dysplasia (FCD) IA (*n* = 3), FCDIIB (*n* = 5), FCDIIIA (*n* = 6), FCDIIIB (*n* = 6), FCD IIID (*n* = 4), and cases with intracranial recording electrode track injuries (*n* = 17)]. Sections from formalin‐fixed paraffin‐embedded tissue blocks, cut at 5 *μ*m, were double‐labeled with combinations of antibodies against PAX6 (1:100; Santa Cruz Biotechnology, Inc., Dallas, TX) and either GFAP (glial fibrillary acidic protein, 1:1500; Dako, Cambridge, UK), nestin (1:6000; Millipore, Watford, UK), calretinin (1:300, Swant, Switzerland), beta‐tubulin (1:700 Sigma Aldrich, Dorset, UK), or doublecortin (1:350, Abcam, Cambridge, UK) using conventional immunohistochemical techniques. Specimens with FCD were classified using the ILAE classification system.^23^ Single‐ and double‐labeled cells were quantified in defined regions‐of‐interest (e.g., around electrode tracts or along cortical layer I/II) using Image Pro Plus (Media Cybernetics, Inc., Rockville, MD). These regions were selected as sites involved with acute or chronic gliosis. The densities of PAX6‐labeled cells were compared between groups using the Kruskal–Wallis test using SPSS v18.0.

## Results

### Subject demographics and properties

Table 1 outlines the characteristics of the subjects with *PAX6* mutations. Mutations identified in the study group fall into four previously defined categories^24^: (1) intragenic mutations leading to predicted premature protein termination, which is likely to lead to nonsense‐mediated decay so that no protein is produced from the mutant allele (*n* = 7); (2) splice site mutants or exon deletions leading to a predicted in‐frame deletion of 36 or 88 amino acids in the paired or PST‐rich transactivation domains, respectively (*n* = 4); (3) mutations causing predicted C‐terminal protein extension that would produce an abnormally elongated protein (*n* = 5); and (4) paired‐domain missense mutations (*n* = 3). Subjects and controls had a median age of 40 years (range 18–73 years) and 32 years (range 23–62 years), respectively. A comparison of the combined location of the mean, and distribution of the ages within both groups, revealed no significant overall difference. There was no significant difference in the mean ICV or sex distribution between groups.

### Whole‐brain analysis

**Figure 1:**
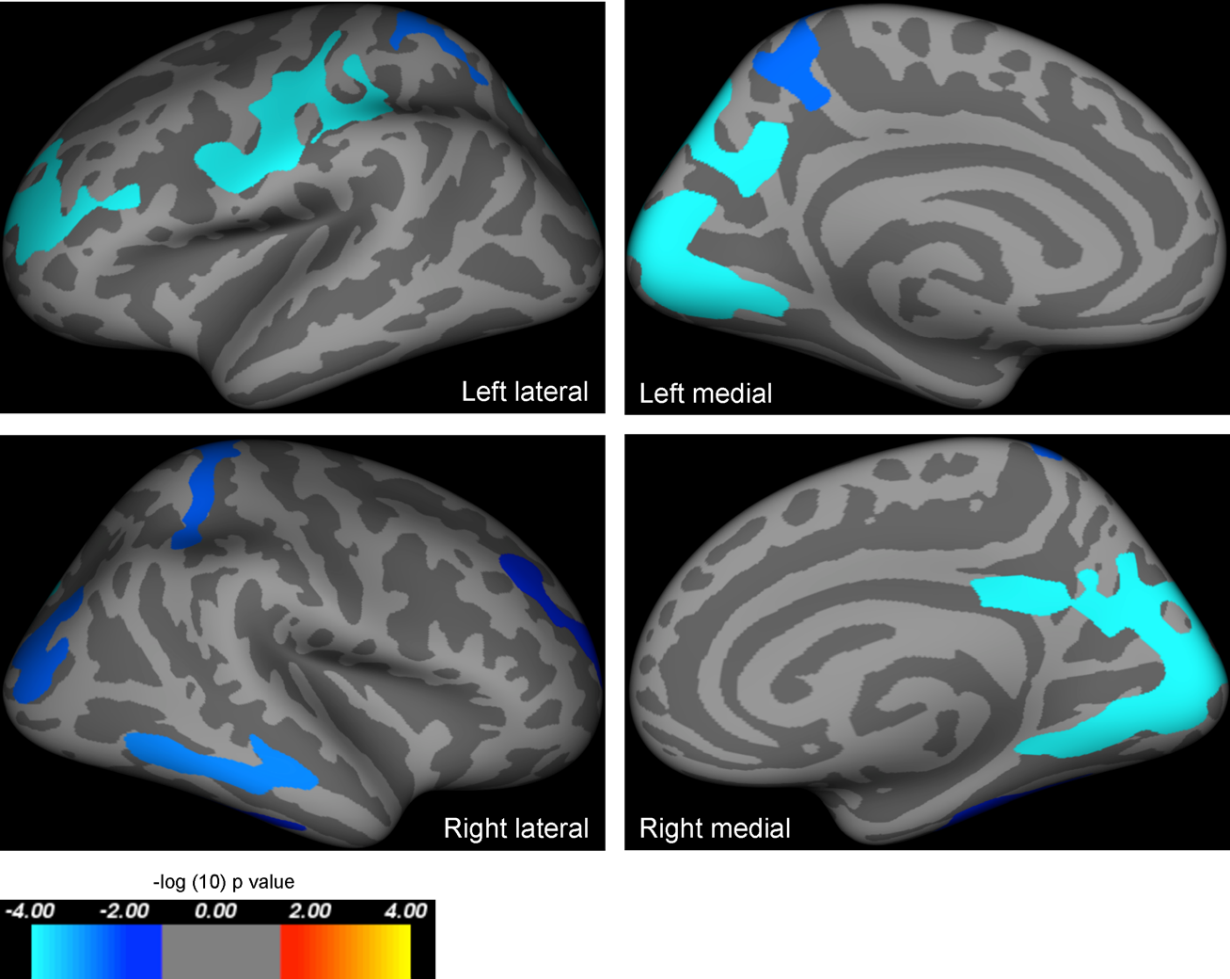
Results of whole‐brain group analysis comparing cortical area in *PAX6* subjects and controls including age and intracranial volume as covariates of no interest. Highlighted regions represent significant reductions in cortical area in *PAX6* subjects compared to controls, and were seen in the calcarine cortex, and parts of the frontal and parietal lobes in both hemispheres. All results are corrected for family wise errors. The *P*‐values of the clusters shown extend from 0.0001 (light blue) to 0.02 (dark blue), and are superimposed on lateral/medial views of the left/right inflated hemispheres derived from the Freesurfer FsAverage brain template made in MNI305 space. Coordinates and *P*‐values for clusters are documented in Table 2.

**Figure 2:**
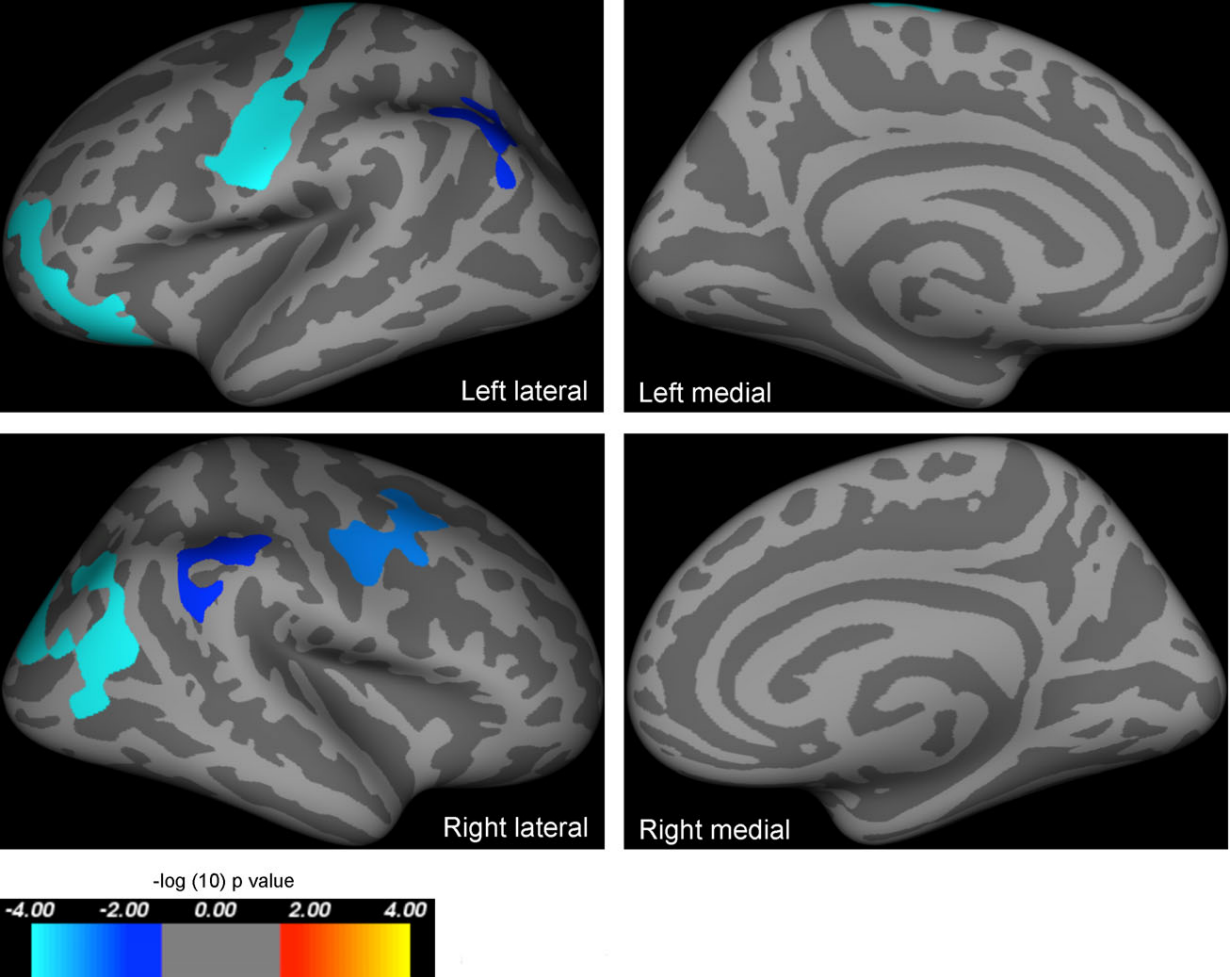
Results of whole‐brain group analysis comparing the correlation between age and cortical thickness in *PAX6* subjects and controls. Highlighted regions represent areas where cortical thickness declined quicker with age in *PAX6* subjects than controls, that is, predominantly in the inferior parietal lobe, and prefrontal and premotor areas in both hemispheres. All results are corrected for family wise errors. The *P*‐values of the clusters shown extend from 0.0001 (light blue) to 0.02 (dark blue), and are superimposed on lateral/medial views of the left/right inflated hemispheres derived from the Freesurfer FsAverage brain template made in MNI305 space. Coordinates and *P*‐values for clusters are documented in Table 3.

Using SBM‐based methods and correcting for age and ICV, there were clusters of significantly reduced cortical area in *PAX6* subjects in the calcarine cortex, precentral and rostral frontal areas, and the superior parietal lobe in both hemispheres (Fig. 1; Table 2). There was a significant interaction between group and age on cortical thickness, with a steeper decline in cortical thickness with age in *PAX6* subjects than in controls. The most significant clusters were found in the inferior parietal, prefrontal, and precentral areas in both hemispheres (Fig. 2; Table 3). The cortical regions of these clusters are considered as the important substrate not only for working memory^25^ but also episodic memory.^26^ There was a borderline positive correlation between age‐corrected digit span and mean cortical thickness across all significant clusters in the left hemisphere (*r*_s_ = 0.542, *P* = 0.030; corrected alpha 0.025, two‐tailed), and a significant correlation in the right hemisphere (*r*_s_ = 0.574, *P* = 0.020; corrected alpha 0.025, two‐tailed). In order to assess the specificity of this correlation, we also carried out a post hoc analysis of the relationship between the thickness of these clusters and verbal fluency and episodic memory. There were positive correlations between episodic memory and mean cortical thickness across all significant clusters in the left (*r*_s_ = 0.715, *P* = 0.002; 2‐tailed) and right hemispheres (*r*_s_ = 0.732, *P* = 0.002; 2‐tailed). There were no significant correlations between cortical thickness and fluency. There was no significant difference in cortical thickness between the groups.

**Table 2:** Results of whole‐brain analysis—clusters of smaller area in *PAX6* subjects compared to controls while including age and intracranial volume as covariates of no interest

Cluster number (hemisphere)	Surface area size (mm^2^)	Talairach coordinates maximum vertex (x, y, z)	Clusterwise probability	Atlas location of maximum vertex
1 (left)	3528.08	−13.4, −91.2, 3.8	0.00010	Pericalcarine
2 (left)	2445.86	−40.8, −11.8, 48.1	0.00010	Precentral
3 (left)	1636.25	−22.1, 41.5, 24.1	0.00010	Rostral middle frontal
4 (left)	1412.04	−7.8, −72.8, 46.2	0.00010	Precuneus
5 (left)	1127.17	−20.6, −43.4, 61.5	0.00280	Superiorparietal
1 (right)	4736.81	14.3, −77.4, 4.8	0.01880	Pericalcarine
2 (right)	1199.94	61.5, −34.9, −14.2	0.00010	Middle temporal
3 (right)	1098.00	35.0, −82.7, 0.6	0.00360	Lateral occipital
4 (right)	1088.29	27.2, −41.2, 52.0	0.02620	Superior parietal
5 (right)	887.52	35.3, −31.9, −22.7	0.00150	Fusiform
6 (right)	849.41	23.5, 42.7, 23.3	0.00380	Rostral middle frontal

**Table 3:** Results of whole brain‐ analysis—clusters of greater decline in cortical thickness with age in *PAX6* subjects compared to controls while including intracranial volume as a covariate of no interest

Cluster number (hemisphere)	Surface area size (mm^2^)	Talairach coordinates maximum vertex (x, y, z)	Clusterwise probability	Atlas location of maximum vertex
1 (left)	2564.12	−36.8, −18.3, 64.5	0.00010	Precentral
2 (left)	1630.18	−38.1, 50.0, −3.4	0.01590	Rostral middle frontal
3 (left)	967.01	−41.3, −67.3, 27.3	0.00010	Inferior parietal
1 (right)	2132.38	34.0, −71.1, 28.7	0.02300	Inferior parietal
2 (right)	1325.30	24.8, 0.1, 46.0	0.00140	Caudal middle frontal
3 (right)	956.65	46.9, −42.5, 37.6	0.00010	Supramarginal

**Figure 3:**
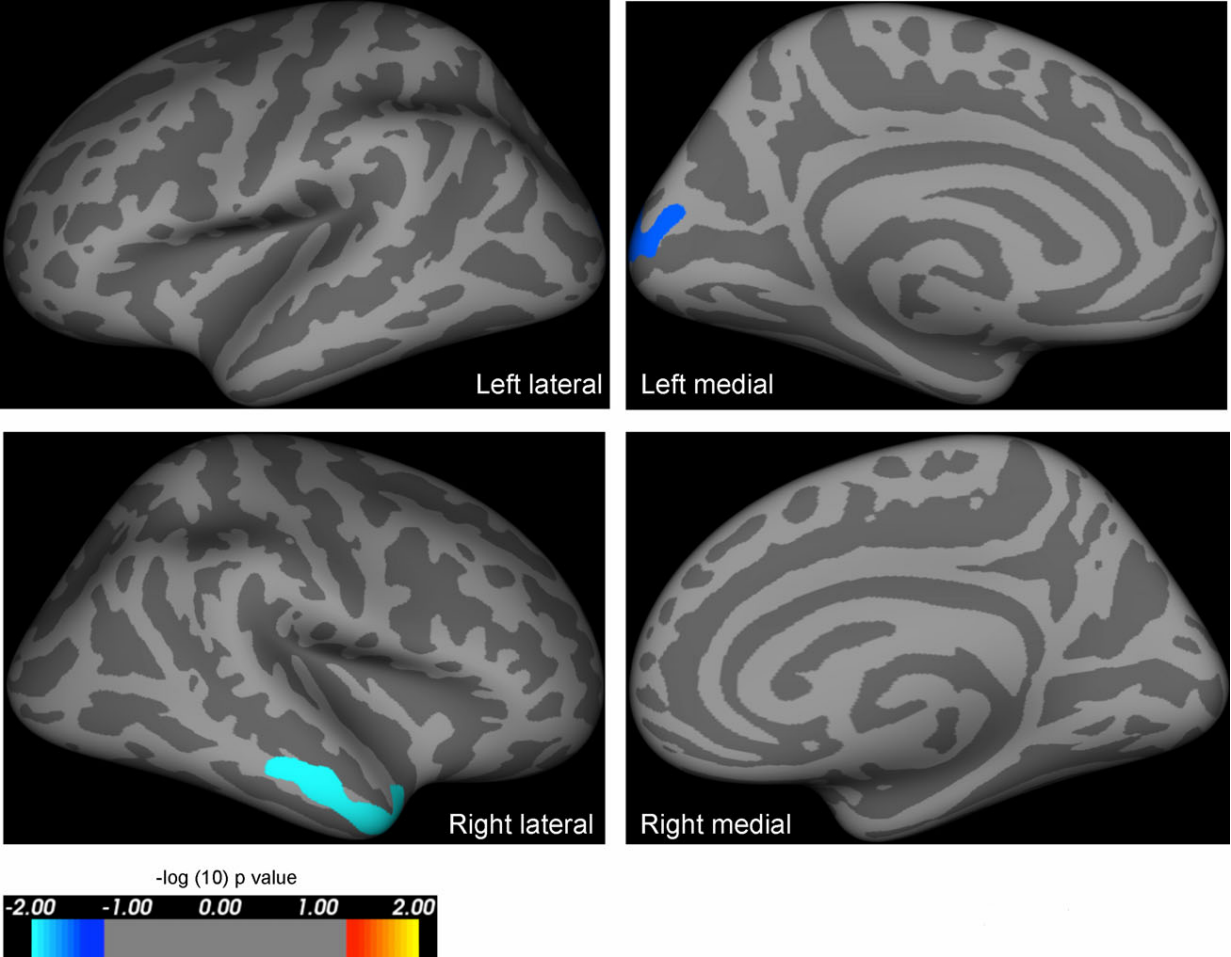
Results of whole‐brain analysis comparing area in mildly affected *PAX6* subjects versus controls, including age and intracranial volume as covariates of no interest. Highlighted regions represent significant reductions in cortical area in *PAX6* subjects compared to controls, and were seen in the medial occipital lobe and middle temporal lobe in mildly affected *PAX6* subjects compared to controls. The reduction in cortical area when compared to controls is less extensive than that seen in severely affected *PAX6* subjects (see Fig. 4). All results are corrected for family wise errors. The *P*‐values of the clusters shown extend from 0.0001 (light blue) to 0.03 (dark blue), and are superimposed on lateral/medial views of the left/right inflated hemispheres derived from the Freesurfer FsAverage brain template made in MNI305 space. The coordinates and *P*‐values of the clusters are documented in Table 4.

**Figure 4:**
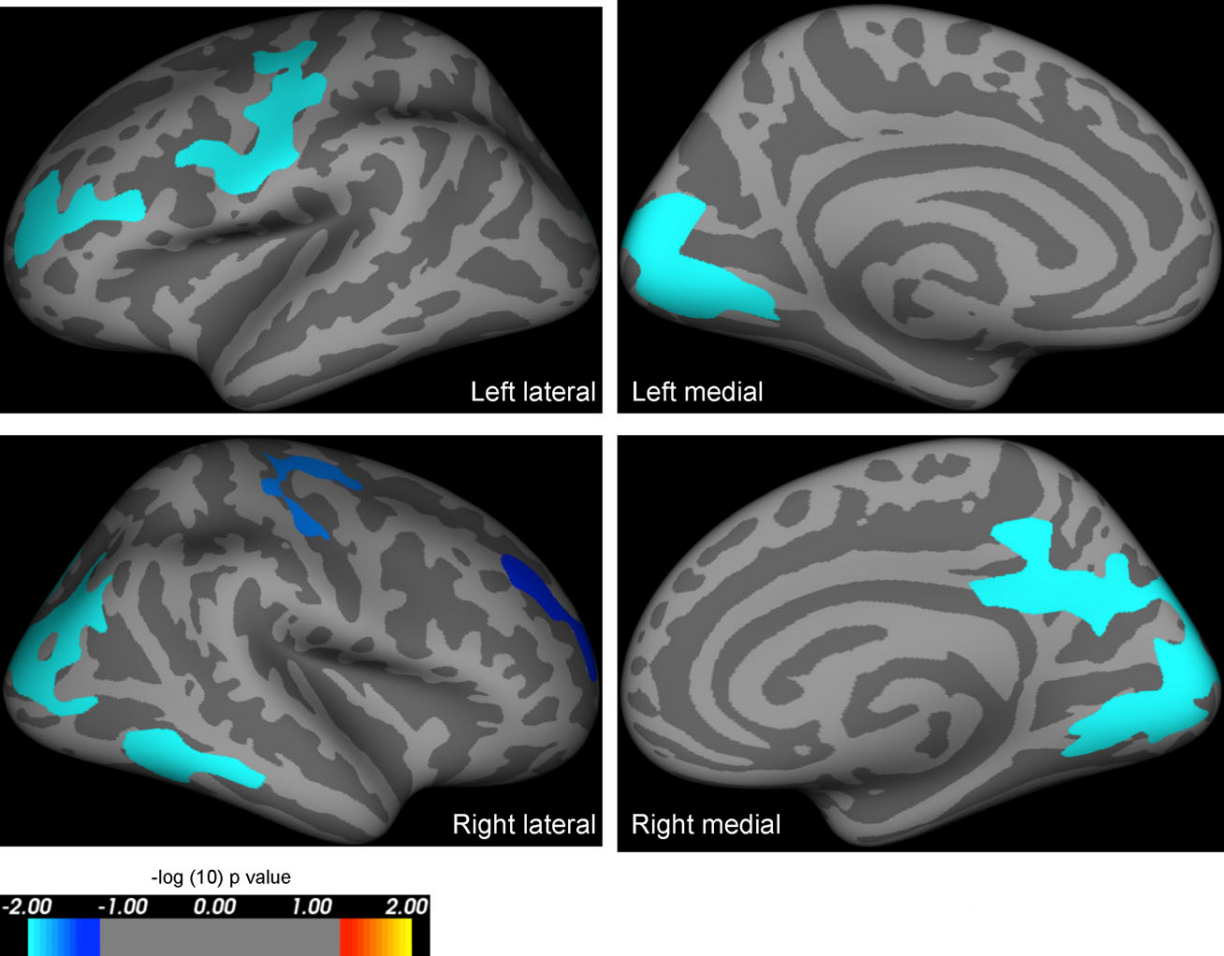
Results of whole‐brain analysis comparing area in severely affected *PAX6* subjects versus controls, including age and intracranial volume as covariates of no interest. Highlighted regions represent significant reductions in cortical area in *PAX6* subjects compared to controls, and were seen in the frontal and occipital lobes in severely affected *PAX6* subjects compared to controls. The reduction in cortical area when compared to controls is more extensive than that seen in mildly affected *PAX6* subjects (see Fig. 3). All results are corrected for family wise errors. The *P*‐values of the clusters shown extend from 0.0001 (light blue) to 0.04 (dark blue), and are superimposed on lateral/medial views of the left/right inflated hemispheres derived from the Freesurfer FsAverage brain template made in MNI305 space. The coordinates and *P*‐values of the clusters are documented in Table 5.

We carried out two further whole‐brain analyses, and an ROI analysis to corroborate these results. First, we repeated the analysis excluding controls under the age of 30 (*n* = 13 remaining) to eliminate group differences in mean age. The results were comparable, with the number and locations of significant clusters being similar to those described above (Tables S1 and S2). Second, we divided *PAX6* subjects into two subgroups. Those with premature protein truncation or C‐terminal extensions tend to have the most severe ocular phenotypes compared to other mutations.^24^ The whole‐brain analysis of area was repeated using these “severe” and “mild” subgroups. Compared to controls using a Bonferroni‐corrected alpha threshold of 0.025, the mild subgroup had less widespread and less significant reductions in whole‐brain cortical area (Fig. 3; Table 4), than the more severe subgroup (Fig. 4; Table 5).

**Table 4:** Results of whole‐brain analysis—clusters of smaller area in “mildly affected” *PAX6* subjects compared to controls while including age and intracranial volume as covariates of no interest

Cluster number (hemisphere)	Surface area size (mm^2^)	Talairach coordinates maximum vertex (x, y, z)	Clusterwise probability	Atlas location of maximum vertex
1 (left)	815.43	−12.9, −96.8, 14.5	0.02600	Lateral occipital
1 (right)	1117.41	62.6, −10.5, −20.4	0.00310	Middle temporal

**Table 5:** Results of whole‐brain analysis—clusters of smaller area in “severely affected” *PAX6* subjects compared to controls while including age and intracranial volume as covariates of no interest

Cluster number (hemisphere)	Surface area size (mm^2^)	Talairach coordinates maximum vertex (x, y, z)	Clusterwise probability	Atlas location of maximum vertex
1 (left)	3382.89	−13.4, −91.2, 3.8	0.00010	Pericalcarine
2 (left)	1552.61	−40.8, −11.8, 48.1	0.00010	Precentral
3 (left)	866.21	−30.3, 46.4, 10.8	0.01690	Rostral middle frontal
1 (right)	6784.06	14.3, −77.4, 4.8	0.00010	Pericalcarine
2 (right)	992.30	61.5, −34.9, −14.2	0.02070	Middle temporal
3 (right)	877.62	27.7, −14.4, 60.2	0.03520	Precentral
4 (right)	804.14	23.5, 42.7, 23.3	0.00810	Rostral middle frontal

### Region‐of‐interest analysis

We also carried out an ROI analysis using atlas‐defined lobar regions (Table 6). After adjusting for age and ICV, there was a significant effect of group [*F*(6,32) = 4.992, *P* = 0.001, Wilks' *λ* = 0.517, partial *η* ^2^ = 0.483] on cortical area. *PAX6* subjects had significantly smaller frontal [*F*(1,37) = 9.397, *P* = 0.004, partial *θ* = 0.203], parietal [*F*(1,37) = 19.623, *P* < 0.001, partial *θ* = 0.347], cingulate [*F*(1,37) = 13.391, *P* = 0.001, partial *θ* = 0.266], and occipital lobar areas [*F*(1,37) = 15.664, *P* < 0.001, partial *θ* = 0.297], but not insular [*F*(1,37) = 0.584, *P* = 0.450, partial *θ* = 0.016] or temporal lobar [F(1,37) = 5.408, *P* = 0.026, partial *θ* = 0.128] areas (corrected alpha 0.008). The *PAX6* mild‐severe ROI analysis corroborated these results and demonstrated a dose‐dependent effect of *PAX6*: after adjusting for age and ICV more severely affected subjects had more widespread reductions in lobar area compared to controls [*F*(12,62) = 2.936, *P* = 0.003, Wilks' *λ* = 0.407, partial *η* ^2^ = 0.362] (Table 7).

**Table 6:** Region‐of‐interest (ROI)‐based estimated combined hemispheric lobar area for *PAX6* patients and controls when corrected for age and intracranial volume (ICV)

Lobe	Group	Mean area (mm^2^)	SE (mm^2^)
Frontal^a^	Controls	36,079	555
	*PAX6*	33,472	600
Cingulum^a^	Controls	3801	84
	*PAX6*	3332	90
Occipital^a^	Controls	11,193	234
	*PAX6*	9771	254
Temporal	Controls	19,326	339
	*PAX6*	18,117	367
Parietal^a^	Controls	24,804	410
	*PAX6*	22,019	444
Insula	Controls	2043	43
	*PAX6*	1993	46

a Indicates significant difference (*P* < 0.008) between patients and controls. SE, standard error.

**Table 7:** Region‐of‐interest‐based estimated combined hemispheric lobar area for “mildly affected” and “severely affected” *PAX6* subjects and controls when corrected for age and intracranial volume (ICV). When the lobar areas were considered separately using planned group contrasts, and a Bonferroni‐adjusted alpha level of 0.008, the only significant difference between mildly affected and control subjects was in the parietal lobe (*P* = 0.001). When controls were compared to severely affected subjects, parietal (*P* = 0.001), occipital (*P* < 0.001), cingulate (*P* = 0.002), and frontal (*P* = 0.009) lobar areas were significantly smaller

Lobe	Group	Mean area (mm^2^)	SE (mm^2^)
Frontal	Controls	36,078	562
	Mild	33,582	977
	Severe^a^	33,408	752
Cingulum	Controls	3801	85
	Mild	3368	147
	Severe^a^	3312	113
Occipital	Controls	11,192	234
	Mild	10,114	406
	Severe^a^	9573	313
Temporal	Controls	19,327	338
	Mild	17,630	588
	Severe	18,399	453
Parietal	Controls	24,805	413
	Mild^a^	21,662	718
	Severe^a^	22,226	553
Insula	Controls	2043	42
	Mild	1900	72
	Severe	2047	56

SE, standard error.

a Indicates significant difference between patients and controls.

**Figure 5:**
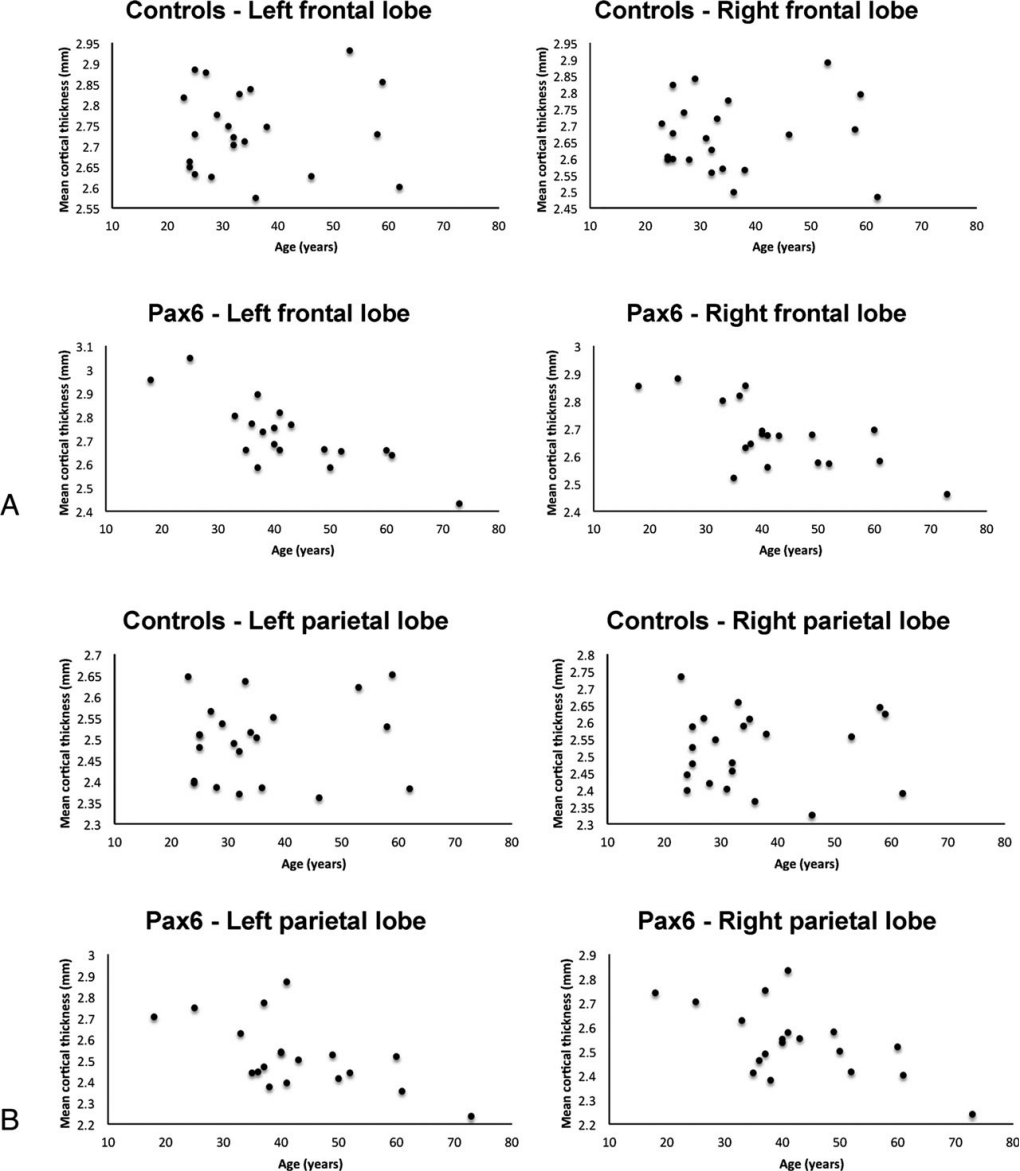
Results of region‐of‐interest analysis. Cortical thickness declines with age in both the frontal and parietal lobes in *PAX6* subjects, but not controls, over the age range sampled. Age was negatively correlated with cortical thickness in (A) the left/right frontal (*r* = −0.792, *P* < 0.001/*r* = −0.705, *P* = 0.001) and (B) the left/right parietal (*r* = −0.634, *P* = 0.002/*r* = −0.640, *P* = 0.002) lobes in *PAX6* subjects only (Bonferroni‐corrected alpha of 0.01).

**Figure 6:**
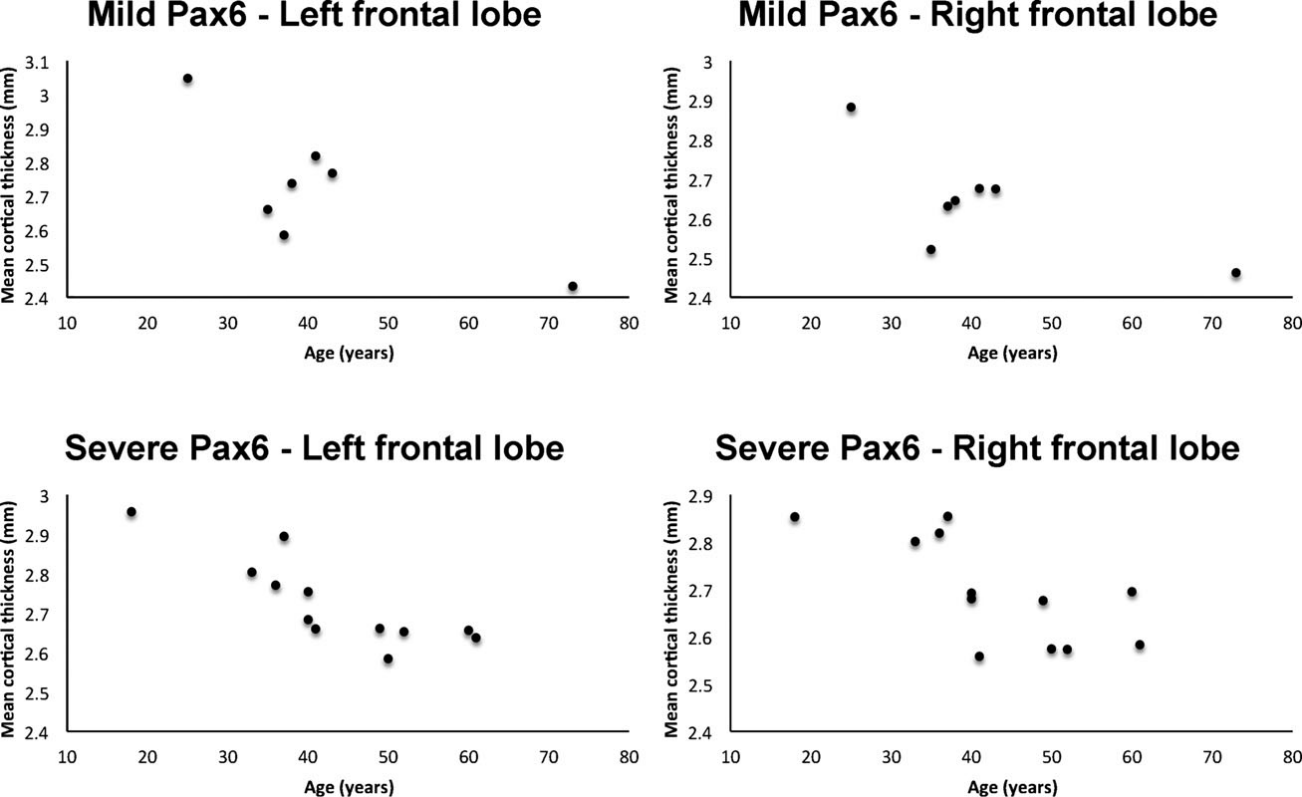
Results of region‐of‐interest analysis showing that cortical thickness declines significantly with age in the frontal lobes in severely affected *PAX6* subjects (bottom row), but not in the mildly affected *PAX6* subjects (top row). Parameter assessments and partial correlations, with a Bonferroni‐corrected alpha of 0.004, show that while controls and mildly affected *PAX6* subjects had no correlation between left or right frontal lobe thickness and age, respectively (top row), a borderline significant negative correlation was present for severely affected *PAX6* subjects (bottom row) (*r* = −0.857, *P* < 0.001 and *r* = −0.755, *P* = 0.004).

There was a significant interaction between the covariates age and group on cortical thickness in the ROI analysis [*F*(6,30) = 2.755, *P* = 0.030, Wilks' *λ* = 0.645, partial *θ* = 0.355]. Using a Bonferroni‐adjusted alpha of 0.008, the group‐by‐age interaction was significant for the frontal lobe [*F*(1,35) = 10.935, *P* = 0.002, partial *θ* = 0.238], and borderline significant for the parietal lobe [*F*(1,35) = 7.814, *P* = 0.008, partial *θ* = 0.183]. In the remaining lobes, there was no significant age‐group interaction with respect to cortical thickness. Further analysis, using a Bonferroni‐corrected alpha of 0.006, revealed that age was negatively correlated with cortical thickness in the left/right frontal (*r* = −0.792, *P* < 0.001/*r* = −0.705, *P* = 0.001) and parietal (*r* = −0.634, *P* = 0.002/*r* = −0.640, *P* = 0.002) lobes in *PAX6* subjects, but not in the left/right frontal (*r* = 0.034, *P* = 0.442; *r* = 0.052, *P* = 0.411) or parietal lobes (*r* = 0.109, *P* = 0.319; *r* = −0.002, *P* = 0.497) of controls (Fig. 5). The *PAX6* mild‐severe ROI analysis corroborated these results, and demonstrated a dose‐dependent effect of *PAX6*. When the results for the combined hemispheric, dependent variables were considered separately using a Bonferroni‐adjusted alpha level of 0.008 the group‐by‐age interaction was borderline significant only for the frontal lobe [*F*(2,32) = 5.354, *P* = 0.01, partial theta = 0.251] such that only severely affected subjects showed a significant decline in cortical thickness with age compared to controls (Fig. 6).

### Tissue analysis

**Figure 7:**
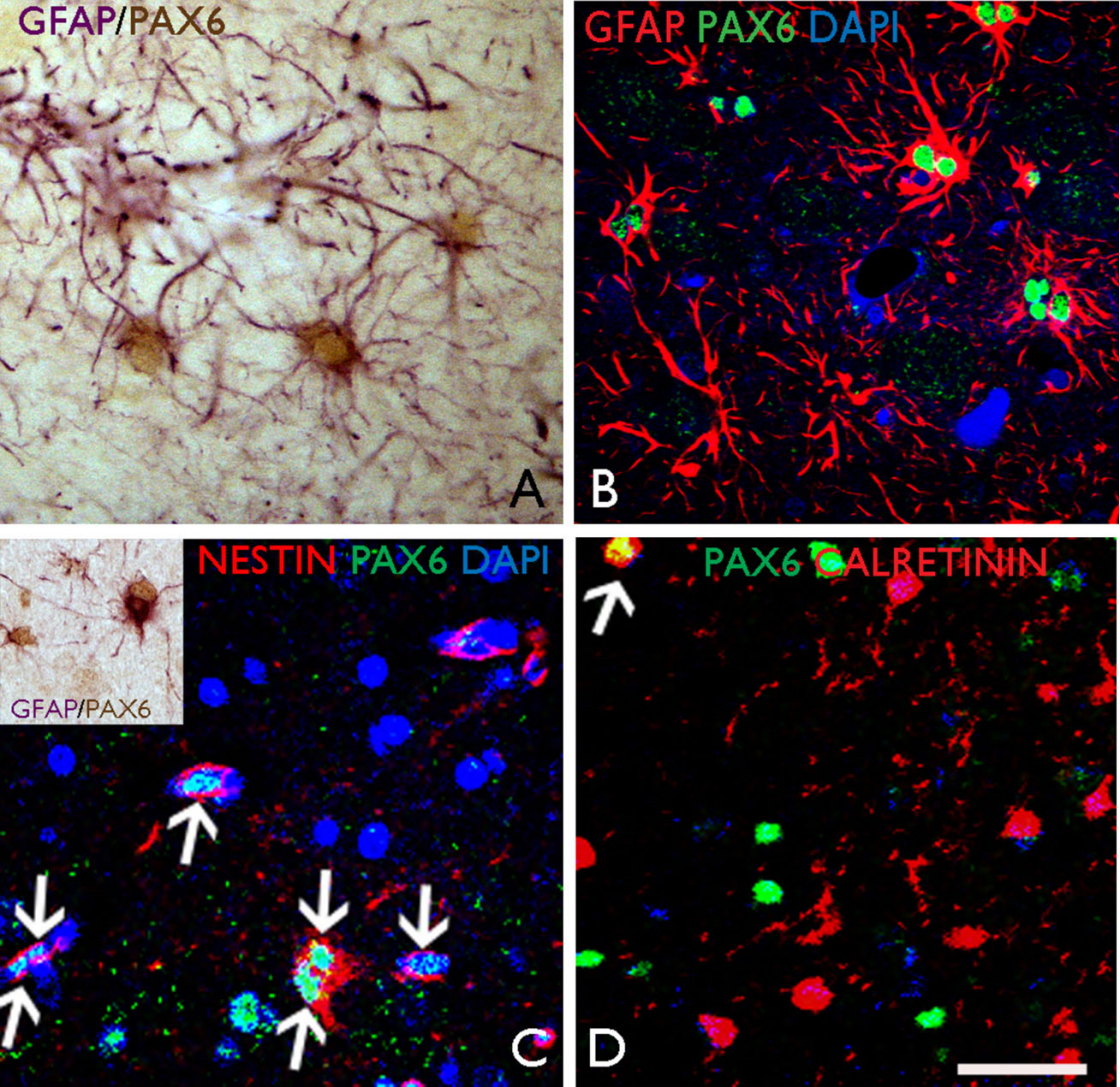
Results of PAX6 immunostaining study. (A) Surgical tissue from an epilepsy surgical sample of temporal lobe cortex with gliosis only. Nuclear labeling is shown for PAX6 in reactive astrocytes in cortical layer I which are GFAP‐positive; (B) Cellular colocalization was demonstrated with confocal laser scanning microscopy (Zeiss LSM610 Meta) for PAX6 and GFAP; (C) In a surgical case of focal cortical dysplasia type IIB, colocalization of PAX6 and nestin is observed in reactive cells (arrows, and a binucleate cell, double arrow); (inset) reactive PAX6‐positive and nestin‐positive glial cells are also observed in the vicinity of an electrode track injury site with reactive gliosis. (D) Confocal imaging demonstrated occasional colocalization between PAX6 and calretinin in the superficial cortex (arrowed cell). Scale bar corresponds to 25 *μ*m in all images; B–D nuclei counterstained with DAPI.

We saw colabeling of PAX6 with GFAP and nestin and to a lesser extent with beta‐tubulin, calretinin and doublecortin, as evidence for expression in both glial and neuronal lineage cells, and in immature and differentiated cell types. PAX6/GFAP double‐labeled cells were numerous in the superficial cortex, white matter, and deeper cortex in all epilepsy cases (Fig. 7). The mean density in the superficial cortex of PAX6/GFAP colabeled cells was 55.6/mm^2^ across all epilepsy cases and controls with no significant difference in densities by underlying pathology. In the gliotic cavities adjacent to electrode track injuries, significant increases in both PAX6/nestin (peak 68.8/mm^2^ in acute injuries) and PAX6/GFAP (peak 69.8/mm^2^ in acute injuries) colabeled cells were noted compared to normal gray and white matter (1.29 and 21.9/mm^2^, respectively) (Fig. 7; *P* < 0.001).

## Discussion

This is the first study is to assess cortical area, and thickness in human heterozygotes with *PAX6* mutations. Using whole‐brain and ROI methods, we have demonstrated exaggerated cortical thinning with age, and abnormalities of cortical patterning in people with heterozygous mutation in *PAX6*. Furthermore, we have demonstrated in patients with *PAX6* mutation, a reduction in cortical thickness that correlates with a decline in working memory in patients, beyond that expected for age. These observations show a genotype effect; those patients with genotypes likely to give rise to less functional protein and more severe ocular phenotypes, appear to have more severe cortical changes. In the second part of this study, we have also demonstrated on‐going expression of PAX6 in adult human cortex in a range of cell types, and dynamic change in PAX6 expression in relation to gliosis and injury. In view of these findings, we hypothesize that *PAX6* may have a role not only in human brain development, but also brain maintenance and resulting function. This has significant implications for our understanding of neurodegenerative disorders: modulation of *PAX6* may offer novel therapeutic strategies.

### Cortical area

Cortical area was reduced in *PAX6* patients primarily in the frontal, parietal, cingulate, and occipital lobes. Animal models of *Pax6* support these findings. Embryonic mouse models have demonstrated that *Pax6* is expressed in a rostrolateral to caudomedial gradient in the telencephalon, and specifies particular rostral and lateral domains such as the primary motor cortex, and somatosensory cortex.^27^ Loss of *Pax6* function in mice results in marked compression of the rostral cortical areas at the level of the motor, cingulate and sensory cortex, accompanied by rostral shift and expansion of the caudal or visual cortex.^3^ Although cortical patterning has not been studied in heterozygous animal models, two studies have assessed the effect of heterozygous *PAX6* mutations on cortical gray matter in humans.^10^, ^28^ However, both studies used VBM‐based methods which conflate area and cortical thickness, and used unmodulated data which makes any data interpretation problematic.^28^ The reduction in frontal and parietal lobe areas seen in this study has therefore not been described before in human heterozygotes. The finding of a reduction in cingulate cortical area has been replicated in both homozygous mouse studies,^29^ and indirectly in VBM‐based studies of human heterozygotes,^28^ and is consistent with an endogenous rostrocaudal expression gradient. Reduced occipital lobe area in *PAX6* patients has also been previously demonstrated indirectly by VBM studies in human heterozygotes,^10^ but is likely to be confounded by the secondary effects of visual impairment in these patients.^30^

### Age‐related decline in cortical thickness

Cortical thickness declines with age at a greater rate in *PAX6* patients compared to controls in the frontal and parietal lobes, and particularly the prefrontal, precentral, and inferior parietal cortex. Given that these areas have been shown to be important in working memory^25^ and given previous findings reported by our group,^11^ we hypothesized and confirmed that the cortical thickness of these areas correlated with age‐corrected working memory. These areas are also important for episodic memory^26^ and a post hoc analysis confirmed that thickness in these areas correlated significantly with episodic memory function, but not other neuropsychological variables. The lethality of the homozygous embryonic mouse models, and paucity of animal heterozygote studies, has resulted in limited investigation into the role of *Pax6* in neuronal maintenance in adult mice. In contrast, there is an increasing body of evidence suggesting that *Pax6* plays an important role in the maintenance of adult corneal and pancreatic tissue. Hart et al. inactivated *Pax6* at 6 months of age in a conditional mouse model to assess the effect of losing *Pax6* function in adulthood.^15^ The effect on glucose homeostasis and the expression of key islet cell markers was measured. Homozygous *Pax6* deletion mice, but not controls, presented with symptoms of diabetes. Immunohistochemical analysis of the pancreas revealed complete loss of Pax6 and reduced expression of insulin, glucagon, and somatostatin. Other markers of islet cell function were also affected. Other studies have shown that *Pax6* is critical to the maintenance and regeneration of the corneal epithelium.^13^ Ouyang et al. showed that this is related to the role of *PAX6* in facilitating the maintenance and differentiation of explanted human limbal stem cells into corneal epithelial cells.^14^ Indeed, heterozygous loss of *PAX6* function is also associated with corneal surface disease in people with aniridia, in whom corneal opacification is frequently observed as transparent epithelial cells normally maintained by limbal stem cells are replaced by opaque conjunctiva‐like cells.^31^

Indirect evidence supporting a maintenance role for *PAX6* in the brain emerges from the increased prevalence of an absent anterior commissure in adults compared to child heterozygotes.^32^ It is also now increasingly appreciated that gene regulatory programs launched early in fetal life to specify neuronal‐type identities, continue to function later in life to maintain postmitotic identities.^33^ Indeed, animal models have demonstrated the persistence of *Pax6* expression,^34^ and its role in the generation of neural stem progenitor cells^35^ and maintenance of neurogenic fate^36^ in the adult rodent brain. Ninkovic et al. demonstrated that ongoing expression of *Pax6* in mature healthy brain is intrinsically required for the survival of dopaminergic neurons in the olfactory bulb through maintenance of crystalline *α*A expression, which prevents caspase‐3‐mediated programmed cell death.^37^ In the absence of *Pax6* function, all dopaminergic olfactory bulb neurons in mature brain died by apoptosis. We previously showed absent or small olfactory bulbs in people with aniridia.^8^ Together, these findings show that *PAX6* is likely to have roles in maintenance of adult human brain structures, as well as their development.

Given the importance of neural stem cells to tissue repair and cognitive performance in aging,^38^, ^39^ and the rostrocaudal distribution of cortical abnormalities seen in nonlethal adult cortex‐specific *Pax6* knockout mice,^40^ it is reasonable to suggest that heterozygous *PAX6* mutation may accelerate age‐related decline because of the normal role of *PAX6* in maintaining neural stem cells and promoting neuronal differentiation.^41^ The role of *PAX6* in maintenance may underlie the faster rate of age‐related frontoparietal cortical thinning seen in this study. Age‐related memory decline has been shown to be aggravated by loss of structural brain integrity in old age,^42^ which may be expected to result in an accelerated decline in working memory beyond expected age‐related changes in adult human *PAX6* heterozygotes. Supporting these findings, previous reports have highlighted deficits in working memory,^11^ and cognitive and behavioral abnormalities related to frontal executive dysfunction in both human *PAX6* heterozygotes^43^ and *Pax6* mouse models.^40^ Ellison‐Wright et al. reported one such family, and demonstrated using fMRI and executive tasks, reduced functional activation in areas similar to those described in this study, namely prefrontal and premotor areas.^28^ Our findings demonstrate a direct physical consequence of reduced *PAX6* function on adult human brain structure, showing dose‐dependency, and underpinning the previous observations.

### Dynamic changes in PAX6 expression in human tissue

Postnatal animal studies have shown that there is strong expression of *Pax6* in neurons in various brain regions, including the olfactory bulb, amygdala, thalamus, and cerebellum. Modest expression is seen in the subgranular zone of the hippocampal dentate gyrus and in the ependymal layer and the subventricular zone of the lateral ventricle, the two areas in which neurogenesis takes place throughout life.^41^ Furthermore, rat models of transient brain ischemia have demonstrated dynamic upregulation of Pax6 expression with reactive astrogliosis, which is thought to represent a protective response to tissue injury.^44^ The results reported here directly demonstrate ongoing expression of *PAX6* in adult cortex in a range of cell types for the first time in human subjects. Dynamic change in *PAX6* expression, in relation to gliosis and injury, was also noted, supporting a role for *PAX6* in adult human cortex in the process of gliogenesis^35^ and in the maintenance of neuronal cells.^34^, ^45^ Recent studies based on murine cell lines^46^ and large‐scale single cell RNA sequencing^47^ have shown that *Pax6* influences the process of neurodegeneration through cascades of genes involved in growth, differentiation, and maturation of neurons and glia, and is an important regulatory mechanism for the maintenance of adult cell‐type identity.

### Limitations

The mean age in the patient and control groups differed, and this may have contributed to differences in cortical parameters between groups. However, age was included as a confounding variable in the analysis of group differences in area, and given that the difference in age versus thickness correlation was the primary finding of interest, it is reassuring to note that there was no statistical difference in the combined location of the mean, and distribution of the ages within both groups. Furthermore, the whole‐brain analysis group results remained similar even when younger control subjects were excluded, such that there was no significant difference in the mean age between groups. Some studies have argued the relationship between cortical parameters and age may vary by location in the brain and may not be consistently linear.^48^ However, the finding that the extent of cortical differences between the groups varied depending on the severity of the *PAX6* genotype suggests that the observed differences are due to genetic factors rather than other variables. Indeed, animal studies have also shown that *Pax6* has dose‐dependent effects.^49^ Furthermore, it is difficult to fit nonlinear models to the data reported here given the small number of subjects. Given the resolution of current MRI techniques, it is also not possible to ascertain the specific biological cause of reduced cortical thickness, which may be due to a variety of factors including neuronal loss, shrinkage of neurons, reductions of synaptic spines, or lower number of synapses.^50^ However, regardless of the downstream biological mechanisms, *PAX6* appears to be important for the maintenance of brain structure, although we could not formally evaluate the developmental effects of *PAX6* mutation that might have confounded some of our findings.

## Conclusion

*PAX6* plays a role in the modulation and progression of cortical volume and function changes in adult life. The importance of *PAX6* for the maintenance of cortical structures has significant implications for their function and for neurodegenerative disorders. Modulation of *PAX6* might in turn offer novel avenues for the treatment of neurodegenerative diseases.

## Conflicts of Interest

The authors declare no conflicts of interest.

## Author Contributions

SMS conceived the study. MY, MM, VVH, and SMS designed and coordinated the study. MY, MM, CV, PJT, JSD, MS, ATM, JL, MT, VVH, and SMS recruited subjects and acquired data. MY, MM, PJT, JL, MT, VVH, and SMS analyzed the data. MY, VVH and SMS prepared the manuscript with input from all co‐authors.

## Supporting information


**Table S1.** Results of whole‐brain analysis—clusters of smaller area in *PAX6* subjects compared to controls over 30 years of age while including age and intracranial volume as covariates of no interest.
**Table S2.** Results of whole‐brain analysis—clusters of greater decline in cortical thickness with age in *PAX6* subjects compared to controls over 30 years of age while including intracranial volume as a covariate of no interest.
