# Supplementary material for: PAX6, brain structure and function in human adults: advanced MRI in aniridia
Source: Ann Clin Transl Neurol. 2016 Apr 12;3(5):314–30. doi: 10.1002/acn3.297 (PMC4863745; doi:10.1002/acn3.297)
Supplement: Supplementary file 1 — Table S1. Results of whole‐brain analysis—clusters of smaller area in PAX6 subjects compared to controls over 30 years of age while including age and intracranial volume as covariates of no interest. Table S2. Results of whole‐brain analysis—clusters of greater decline in cortical thickness with age in PAX6 subjects compared to controls over 30 years of age while including intracranial volume as a covariate of no interest. [file ACN3-3-314-s001.doc]

Supplementary Table 1 – Results of whole brain analysis - clusters of smaller area in *PAX6* subjects compared to controls over 30 years of age whilst including age and intracranial volume as co-variates of no interest

| **Cluster number (hemisphere)** | **Surface area size (mm^2^)** | **Talairach coordinates maximum vertex (x,y,z)** | **Clusterwise probability** | **Atlas location of maximum vertex** |
| --- | --- | --- | --- | --- |
| 1 (left) | 3976.88 | -9.9 -83.1 3.6 | 0.00010 | pericalcarine |
| 2 (left) | 2057.73 | -33.6 -15.2 36.1 | 0.00010 | precentral |
| 3 (left) | 1022.23 | -46.9 17.8 9.1 | 0.00580 | pars opercularis |
| 4 (left) | 962.13 | -31.3 -86.1 12.6 | 0.00890 | lateral occipital |
| 5 (left) | 1316.23 | -6.1 -58.7 31.7 | 0.00080 | precuneus |
| 6 (left) | 1840.45 | -16.5 -61.9 57.5 | 0.00010 | superior parietal |
| 7 (left) | 1390.48 | -20.8 44.7 27.8 | 0.00030 | rostral middle frontal |
| 1 (right) | 4434.30 | 11.0 -82.3 32.0 | 0.00010 | superior parietal |
| 2 (right) | 892.99 | 10.8 19.5 -14.3 | 0.01820 | medial orbito-frontal |
| 3 (right) | 1140.91 | 55.9 -37.9 -10.1 | 0.00270 | middle temporal |
| 4 (right) | 923.72 | 29.1 -79.2 10.0 | 0.01350 | lateral occipital |
| 5 (right) | 1096.90 | 28.1 -40.0 54.9 | 0.00370 | superior parietal |
| 6 (right) | 844.15 | 53.0 2.3 -7.9 | 0.02740 | superior temporal |
| 7 (right) | 931.80 | 42.2 -14.3 20.8 | 0.01280 | post central |

Supplementary Table 2 – Results of whole brain analysis – clusters of greater decline in cortical thickness with age in *PAX6* subjects compared to controls over 30 years of age whilst including intracranial volume as a co-variate of no interest

| **Cluster number (hemisphere)** | **Surface area size (mm^2^)** | **Talairach coordinates maximum vertex (x,y,z)** | **Clusterwise probability** | **Atlas location of maximum vertex** |
| --- | --- | --- | --- | --- |
| 1 (left) | 2101.72 | -24.4 -19.1 58.8 | 0.00010 | precentral |
| 2 (left) | 820.26 | -44.0 -52.5 38.5 | 0.04690 | inferior parietal |
| 1 (right) | 1259.28 | 39.0 5.4 39.2 | 0.00330 | caudal middle frontal |
